# The effectiveness of exercise interventions in the improvement of sleep in older adult people: a meta-analysis

**DOI:** 10.3389/fpubh.2025.1529519

**Published:** 2025-03-05

**Authors:** Di Geng, Xiaogang Li, Guotao Sun

**Affiliations:** ^1^Department of Physical Education, University of Electronic Science and Technology of China, Chengdu, China; ^2^School of Physical Education, Sichuan Normal University, Chengdu, China; ^3^College of Education and Sports Science, Yangtze University, Jingzhou, China

**Keywords:** meta-analysis, exercise, physical activity, sleep, older people

## Abstract

**Background:**

Sleep problems are prevalent among the older adult population, with a significant impact on their health and overall well-being. Several randomized controlled trials (RCTs) have yielded controversial results regarding the efficacy of exercise interventions in the improvement of sleep among older adult people. This systematic review and meta-analysis aim to assess the influence of exercise interventions on sleep quality within this demographic.

**Methods:**

A search was conducted across four databases, namely Web of Science, PubMed, Embase, and SportDiscus, in order to identify randomized controlled trials investigating exercise interventions and sleep in the older adult. The quality of the studies included was evaluated by two researchers according to the PEDro scale. Meta-analysis and sensitivity analysis were performed utilizing RevMan 5.4 and Stata 17 software.

**Result:**

A total of fifty studies encompassing 3,937 participants were included in the analysis. Regarding patient-reported sleep parameters, exercise interventions exhibited enhancements in sleep quality (WMD = −2.18, 95%CI: −2.83 to −1.53, *p* < 0.01) and reductions in insomnia severity (SMD = −0.52, 95%CI: −0.79 to −0.25, *p* < 0.01), albeit without significant improvements in daytime sleepiness (SMD = −0.66, 95%CI: −1.41 to 0.09, *p* = 0.09). In terms of clinician-reported sleep parameters, exercise interventions resulted in increased total sleep time (WMD = 8.98, 95%CI: 1.19 to 16.78, *p* < 0.05) and sleep efficiency (WMD = 3.66, 95%CI: 2.46 to 4.85, *p* < 0.01), and reduced wake time after sleep onset (WMD = −11.85, 95%CI: −15.58 to −8.11, *p* < 0.01), but did not decrease sleep onset latency (WMD = −3.05, 95%CI: −6.23 to 0.13, *p* = 0.06) or the number of awakenings during sleep (WMD = −0.73, 95%CI: −1.98 to 0.52, *p* = 0.25).

**Conclusion:**

Exercise interventions have demonstrated positive effects on enhancing sleep quality among the older adult population. This study lends support to the utilization of exercise interventions as a safe, feasible, and effective non-pharmacological treatment approach for enhancing sleep among older individuals.

**Systematic review registration:**

https://www.crd.york.ac.uk/PROSPERO/view/CRD42024530227, Identifier CRD42024530227.

## Introduction

Sleep, as a crucial restorative behavior, has a profound influence on individual’s health and well-being (1). As a sensitive indicator of individual health, sleep is closely linked to both physical and mental health, as well as several key psychological and behavioral structures. Sleep of high quality is essential for health, learning, memory, and energy, playing critical roles in physiological restoration, cognitive enhancement, and immune function regulation (2, 3). However, sleep problems have become a global public health concern, affecting individuals worldwide. According to data from the Centers for Disease Control and Prevention in the United States, approximately one-third of adults experience sleep disorders, with higher prevalence rates observed among the older adult demographic compared to their younger counterparts (4). As individuals age, sleep patterns gradually change, characterized by diminished sleep duration and efficiency, heighted sleep fragmentation, decreased duration of rapid eye movement (REM) and slow-wave sleep (5). Additionally, older adult people frequently suffer from chronic systemic diseases such as hypertension, diabetes, and rheumatism, the symptomatic manifestations of which can compromise sleep quality (6). All these factors contribute to poorer sleep condition among older adult population compared to younger individuals. Epidemiological studies indicate that more than half of older adult individuals report sleep-related complaints, manifesting as difficulties initiating sleep, poor sleep continuity, premature awakening, and diurnal somnolence (7, 8). Prolonged sleep disturbances can exacerbate physiological and pathological aging among the older adult, raising the likelihood of diseases such as Alzheimer’s, stroke, and atherosclerosis (9–11), in addition to anxiety, depression, and other psychological disorders (12). How to effectively prevent sleep problems in older adult people and improve their sleep have garnered widespread clinical attention.

In clinical practice, strategies aimed at optimizing sleep quality among the older adult encompass both pharmacological and non-pharmacological treatments. Commonly used medications for pharmacological treatment include sedative-hypnotic drugs, atypical antipsychotics, antidepressants, and melatonin, along with melatonin receptor agonists (13–16). In spite of the short-term improvement in sleep quality among the older adult population, prolonged pharmacological intervention is discouraged owing to the attendant risks of tolerance and dependence (17). Furthermore, the majority of pharmacological treatments are associated with the risk of cognitive and behavioral changes, including memory loss, dizziness, or loss of balance leading to falls (18). Non-pharmacological interventions refer to methods of improving sleep that do not involve medication. Compared to pharmacological interventions, non-pharmacological therapies may have longer-lasting effects and lower risk of adverse events, rendering them preferable for older adult individuals experiencing sleep disturbances (19, 20). The most common is psychotherapy, with cognitive-behavioral therapy and mindfulness-based stress reduction therapy included, both of which have been proven to have positive impact on sleep quality among the older adult demographic (21, 22). Nevertheless, psychotherapy requires a longer duration for sleep improvement, coupled with the necessity for administration by trained therapists, rendering it costly and less accessible to a large number of patients (23). Therefore, it is indispensable to explore simpler and cost-effective non-pharmacological treatment options.

Exercise therapy is a non-pharmacological treatment option, in the form of exercise prescriptions for patients or sub-healthy populations, offering advantages such as minimal side effects, wide accessibility, and lower investment costs (24). Some studies have investigated the relationship between exercise and sleep in the older adult population. Epidemiological research indicates that regular exercise is connected with better self-reported sleep quality among older adult people (25), while less physical activity may lead to insomnia in later life (26). Additionally, several RCTs have examined the effects of exercise therapy on sleep among older adult individuals, but inconsistent results exist possibly due to differences in measurement tools, sample characteristics, and other factors. Noteworthy are the contradictory conclusions drawn in the two recent studies. One examined the effects of a 12-week exercise program on sleep quality in older adult people, showing improvements in subjective sleep quality and clinician-reported sleep parameters compared to a placebo control group (27). Another found that a 12-week exercise intervention did not improve sleep quality significantly in older adult community residents compared to routine care controls (28). These conflicting findings pose challenges for clinical practice.

In recent years, several systematic or narrative reviews have included discussions on the relationship between exercise and sleep, whose conclusions suggest that exercise therapy has a positive impact on self-reported sleep quality in adults to certain degree, particularly on sleep latency and efficiency (29, 30). Nonetheless, most of these studies focused on relatively younger adults, therefore these findings may not apply to older populations. Due to prevalent chronic diseases among older adult population as well as their declining physical capabilities, exercise regimens designed for general adults may not be suitable for the older adult. Recently, a systematic review evaluated the effects of physical exercise on sleep in older adult people (31). Although it demonstrated positive effects of exercise programs on various aspects of sleep in this population, only six of the included studies were RCTs. Given the limited trials, small sample sizes, and the interventions used predominantly involving mind–body exercises such as yoga, Tai Chi, and Baduanjin, the overall impact of exercise intervention on sleep remains unclear. Furthermore, new evidence from additional RCTs has emerged since these studies were published. Therefore, there is a need to update and synthesize the existing evidence to further confirm the relationship between exercise therapy and sleep in older adult population. The primary objective of the present study is to conduct a systematic review and meta-analysis to evaluate the effectiveness of exercise therapy in the improvement of sleep quality among older adult individuals and to evaluate whether and how exercise interventions may improve patient-reported or clinician-reported sleep outcomes among this demographic cohort.

### Method

To ensure methodological rigor and scientific integrity, this meta-analysis strictly adhered to the Preferred Reporting Items for Systematic Reviews and Meta-Analyses (PRISMA) statement in both the conduct of the analysis and the writing of the report (32). The protocol for this review has been registered on the PROSPERO platform^1^ under registration number CRD42024530227.

### Eligibility criteria

This study established eligibility criteria based on the evidence-based medicine PICOS framework (33).

Participant: We included studies focusing on older adult people with a mean age of 60 years or above, regardless of their diagnostic status regarding sleep disorders. In other words, no specific baseline sleep condition threshold was imposed, whereas participants with acute illnesses were excluded. Intervention: The intervention was a pre-determined regular exercise program, including exercises of any mode, intensity, duration, and frequency. Studies combining exercise therapy with non-exercise interventions were excluded due to the challenge in isolating the effect of exercise on sleep in older adult people. Comparison: We included studies comparing exercise interventions with no additional exercise or physical activity in the control group. Control conditions may include no intervention, placebo, usual care, waitlist, health education, etc. Outcome: Included studies reported sleep-related outcomes pre-and post-intervention, providing sufficient statistical data (sample size, mean, standard deviation, or standard error). The measurement tools employed in the studies were not restricted, ranging from standardized scales such as the Pittsburgh Sleep Quality Index (PSQI), Epworth Sleepiness Scale (ESS), Athens Insomnia Scale (AIS), Insomnia Severity Index (ISI), as well as objective measurement instruments like polysomnography (PSG), actigraphy, etc. Study design: To obtain high-level evidence, only randomized controlled trials (RCTs) were included, thereby reducing heterogeneity across studies and enhancing the validity of combined results. These studies were sourced from peer-reviewed English journals. Additionally, studies manifesting significant baseline disparities between the exercise intervention group and the control group were excluded.

### Search strategy

We conducted searches in four electronic databases—Web of Science, PubMed, Embase, and SPORTDiscus—to identify studies on the impact of exercise interventions on sleep in older adult people. The search period spanned from the inception of each database to October 24, 2024. The initial search employed four key terms: aged or older adult, exercise or physical activity, sleep or sleep quality, and randomized controlled trials. Following the retrieval strategies of previous relevant reviews, search keywords were designed for each major term. Additionally, based on guidance from experienced librarians, we combined subject headings and free terms corresponding to the search keywords from the database thesauri to maximize the retrieval of relevant records.

### Literature selection

The study selection process involved three steps: First, EndNote X9 literature management software was adopted in order to merge search results from each database and remove duplicate records of the same report. Second, titles and abstracts of the remaining reports were reviewed to eliminate off-topic reports. Finally, the full text of potentially relevant reports was obtained and examined to assess their eligibility for inclusion. Two members of the systematic review team (DG and XL) participated in the screening of titles and abstracts, followed by independent full-text reviews of potentially relevant reports. In cases where disagreements could not be resolved through discussion, arbitration was provided by the team leader (GS). In cases of multiple study reports based on identical sample, only the most comprehensive report or the study with the largest sample size was included.

### Data extraction

To ensure data accuracy and minimize potential bias, data extraction was performed independently by two experienced members (DG and XL) in a double-blind manner. A pre-designed electronic spreadsheet facilitated meticulous entry of extracted data, including: (1) literature information (author names, publication year, country/region); (2) participant details (source, sample size, age); (3) intervention details (type, duration per session, frequency, duration); (4) control conditions; and (5) sleep-related index measurement tools and outcomes (mean, standard deviation). In cases where studies lacked sufficient information, authors were contacted via email for clarification. Any disagreement during data extraction that could not be resolved through discussion was arbitrated by GS.

### Quality assessment of the selected studies

This study utilized the Physiotherapy Evidence Database (PEDro) scale to assess the included literature (34). The PEDro scale is specifically designed to evaluate the methodological quality of randomized controlled trials in physiotherapy, with reliability and validity (35). It consists of 11 criteria, such as participant eligibility criteria, random allocation, concealment of allocation, baseline comparability, blinding (participants, therapists, and assessors), attrition rate (<15%), intention-to-treat analysis, between-group comparisons, and point and variability measures. The first criterion is not scored, and the remaining criteria are each scored as 1 point, contributing to a total score of 10. One point is achieved for meeting each criterion and 0 point for failing to do it. However, a review has highlighted that blinding participants and therapists may often be infeasible in many exercise intervention trials. Consistent with Liang et al., we categorized the quality of the included studies into three levels: high quality (score ≥ 6), adequate quality (score 4–5), and low quality (score ≤ 3) (36). Two team members (DG and XL) scored the included literature independently based on the assessment criteria. Any disagreements in quality assessment that could not be resolved through discussion were arbitrated by GS.

### Statistical analysis

To explore the impact of exercise interventions on sleep quality among elder individuals, this meta-analysis adopted Review Manager 5.4 software to statistically combine results from multiple independent studies. We utilized post-intervention outcome indicator data (sample size, mean, standard deviation) from intervention and control groups. In studies where standard errors were provided, we calculated standard deviations by multiplying the standard error by the square root of the sample size. In cases where measurement units were consistent across the studies included, we used the weighted mean difference (*WMD*) as the effect measure. For studies with different measurement units or methods, the standardized mean difference (*SMD*) served as the effect measure. When aggregating the effect sizes from individual studies, we employed the inverse variance method to determine the weight assigned to each study. The threshold for statistical significance of combined effect sizes was set at *p* < 0.05. The heterogeneity of included studies was assessed using Q-test and *I^2^* statistic values. The *I^2^* value represents the degree of heterogeneity, where 25, 50, and 75% correspond to low, moderate, and high heterogeneity, respectively (37). If *I^2^* ≥ 50%, a random-effects model was utilized to calculate combined effect sizes and 95% confidence intervals (CIs); a fixed-effects model was adopted otherwise. To explore potential sources of heterogeneity, subgroup analysis was conducted based on participant sources, control types, and study quality. Sensitivity analysis was conducted using the one-by-one removal method to evaluate the influence of each study on the overall results. When the number of included studies was ≥10, a funnel plot was generated using Review Manager 5.4 to visually assess publication bias. Additionally, Egger’s regression test was performed using Stata 17.0 to detect significant publication bias (*p* < 0.05). Given the aim of investigating the impact of exercise interventions on sleep in older individuals, in cases where two or more subgroups were included in a study, they were merged into a single exercise intervention group for comparison with the control group. The formula for the combination of subgroup data is as follows: $M=\frac{N_{1}\times M_{1}+N_{2}\times M_{2}}{N_{1}+N_{2}}$ , $SD=\sqrt{\frac{\left(N_{1}-1\right)\times SD_{1}^{2}+\left(N_{2}-1\right)\times SD_{2}^{2}+\frac{N_{1}\times N_{2}}{N_{1}+N_{2}}\times \left(M_{1}^{2}+M_{2}^{2}-2\times M_{1}\times M_{2}\right)}{N_{1}+N_{2}-1}}$ , where *M* represents the mean, *N* represents the sample size, and *SD* represents the standard deviation (38).

## Results

### Literature search

**Figure 1:**
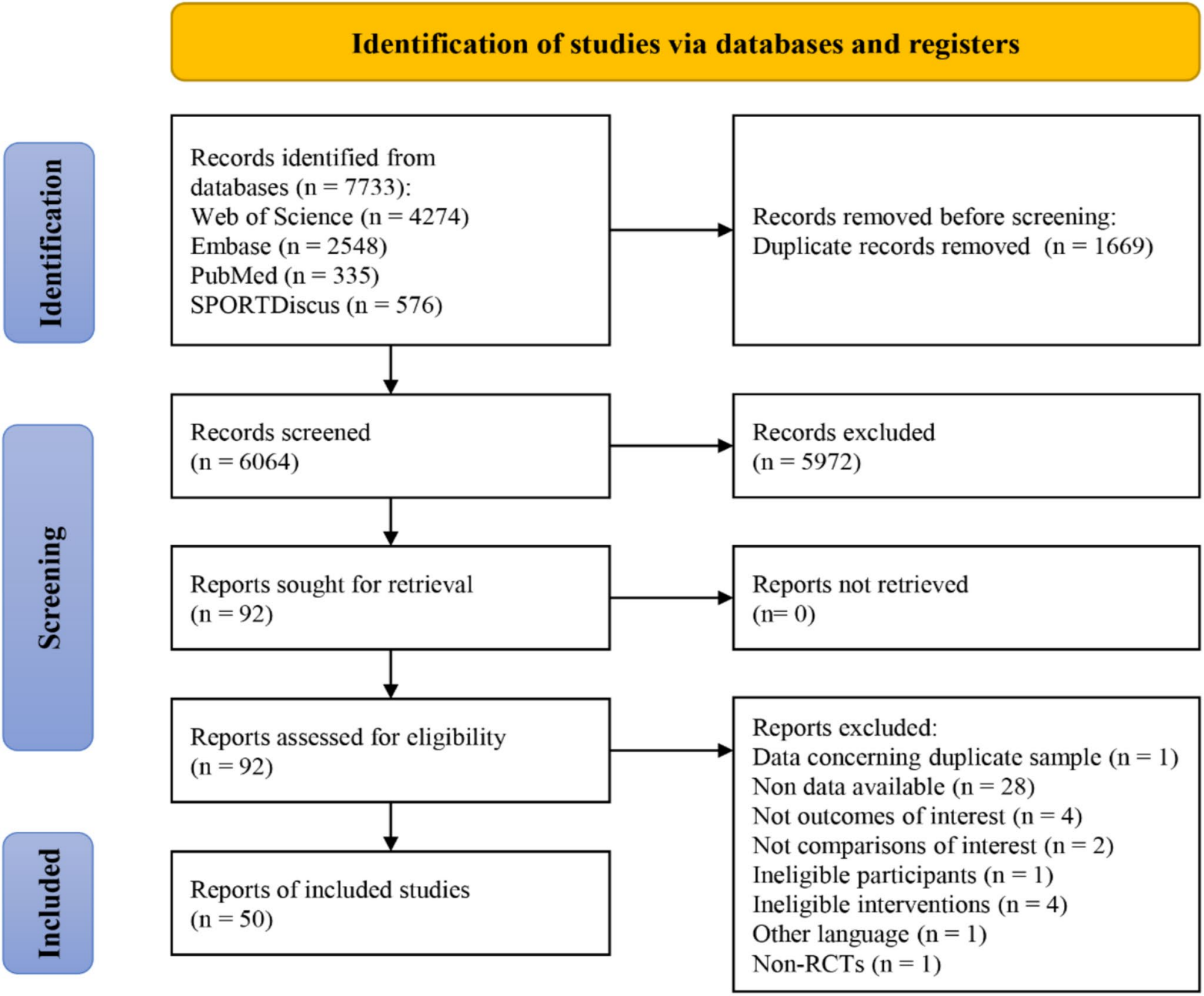
A flow diagram of the search results and study selection.

This study searched four databases, yielding a total of 7,733 records. After removing duplicates utilizing EndNote X9 literature management software, 6,064 records remained. Initial screening based on titles and abstracts led to the exclusion of 5,972 irrelevant records. Subsequently, full-text screening of the remaining 92 records resulted in the exclusion of 42 articles for various reasons: data duplication (*n* = 1), unavailable data (*n* = 28), non-matching control conditions (*n* = 2), non-English language (*n* = 1), non-RCTs (*n* = 1), outcome indicators mismatch (*n* = 4), intervention content mismatch (*n* = 4), and ineligible subject (*n* = 1). Ultimately, 50 studies (27, 28, 39–86) were included in the meta-analysis. The literature screening process is illustrated in Figure 1.

### Study characteristics

The basic characteristics of the included studies are summarized in Table 1. This study encompassed 50 trials conducted in various countries and regions including the United States, United Kingdom, Germany, Mainland China, Taiwan, Hong Kong, Tunisia, Brazil, Spain, Thailand, Turkey, Saudi Arabia, South Korea, India, Vietnam, and Iran. These studies collectively recruited 3,937 participants from community settings, nursing homes, public health centers, outpatient clinics, and hospitals. The participant populations encompassed a diverse range, including general older adult people, individuals with muscular dystrophy, metabolic syndrome, knee osteoarthritis, stroke, chronic insomnia, mild cognitive impairment, depression, sleep disorders, postmenopausal women, heart failure patients, colon cancer patients, lung cancer patients, individuals with memory complaints, Alzheimer’s disease patients.

**Table 1:** Characteristics of eligible studies.

Study (Year)	Country/Region	Source of participants	Participants size	Age of participants	Intervention		Frequency and period	Outcome measures
					E	C		
Wang et al. (39)	China Mainland	General older adult	60	67.55 ± 4.98	Tai Chi	Routine activities	60 min/session, 3 session/weeks, 8 weeks	PSQI, ESS, ISI, PSG
Song et al. (40)	China Mainland	Mild cognitive impairment	89	75.97 ± 6.31	Dancing exercise	Health education	60 min/session, 3 session/weeks, 16 weeks	PSQI
Sánchez-Alcalá et al. (41)	Spain	Mild cognitive impairment	92	71.83 ± 2.96	Dancing exercise	Normal lifestyle	60 min/session, 2 session/weeks, 12 weeks	PSQI
Li et al. (42)	China Mainland	General older adult	48	67.58 ± 4.65	Tai Chi	Routine activities	60 min/session, 3 session/weeks, 8 weeks	PSQI, ISI, actigraphy
He et al. (43)	Hong Kong	Sleep disturbances	26	69.74 ± 4.84	Tai Chi	Routine activities	60 min/session, 3 session/weeks, 4 weeks	ISI, actigraphy
Teruel-Hernández et al. (44)	Spain	Dementia	20	70.05 ± 7.32	cardiovascular exercise	Sleep recommendations	60 min/session, 3 session/weeks, 20 weeks	PSQI, ESS
Tung et al. (45)	Taiwan	Probable sarcopenia	103	79.53 ± 8.25	Acupunch exercise	Usual care	40 min/session, 3 session/weeks, 6 months	PSQI
Baklouti et al. (46)	Tunisia	General older adult	160	65–85	Yoga	Routine activities	80 min/session, 2 session/weeks, 2 months	PSQI
Zhou et al. (47)	China Mainland	Metabolic syndrome	83	78.99 ± 9.93	Aerobic and resistance training	Routine activities	50 min/session, 3 session/weeks, 12 weeks	PSQI
de Sá Souza et al. (27)	Brazil	Sarcopenia	28	76.03 ± 6.73	Resistance training	Weekly meetings	3 session/weeks, 12 weeks	PSG, ESS, PSQI, ISI
Song et al. (48)	China Mainland	Knee osteoarthritis	40	64.15 ± 8.43	Tai Chi	Health education	60 min/session, 3 session/weeks, 12 weeks	PSQI
Marupuru et al. (28)	America	Stroke survivors	145	69.97 ± 10.07	Tai Chi and strength	Usual care	60 min/session, 3 session/weeks, 12 weeks	PSQI
Siu et al. (49)	Hong Kong	Chronic insomnia	320	67.28 ± 6.88	Tai Chi and traditionnal exercise	Usual care	60 min/session, 3 session/weeks, 12 weeks	PSQI, ISI, actigraph
Li et al. (50)	China Mainland	Cognitive impairment	59	72.17 ± 7.61	Resistance training	Routine activities	60 min/session, 3 session/weeks, 12 weeks	Actigraph
Shree Ganesh et al. (51)	India	General older adult	96	64.05 ± 3.92	Yoga	Non-intervention	3 session/weeks, 3 months	PSQI
de Lima et al. (52)	Brazil	General older adult	29	67.59 ± 5.21	Xbox Kinect exercise	Non-physical activity	60 min/session, 3 session/weeks, 6 weeks	PSQI
Jiménez-García et al. (53)	Spain	General older adult	73	68.49 ± 5.18	Interval training	Normal lifestyle	45 min/session, 2 session/weeks, 12 weeks	PSQI
Wang et al. (54)	China Mainland	Cognitive impairment	111	68.31 ± 5.19	Structured limbs-exercise	Healthy education	60 min/session, 3 session/weeks, 24 weeks	PSQI
Tseng et al. (55)	Taiwan	General older adult	40	61.65 ± 7.04	Walking and stretching exercises	Sleep hygiene education	50 min/session, 3 session/weeks, 12 weeks	PSQI, actigraph
Phansuea et al. (56)	Thailand	Moderate depression	66	70 ± 6.63	Qigong	Singing and praying activities	60 min/session, 3 session/weeks, 12 weeks	PSQI
Fan et al. (57)	China Mainland	Sleep disturbances	139	71.1 ± 6.3	Baduanjin	Normal lifestyle	45 min/session, 5 session/weeks, 24 weeks	PSQI
Song et al. (58)	China Mainland	Cognitive impairment	120	75.78 ± 6.28	Aerobic exercise	Health education	60 min/session, 3 session/weeks, 16 weeks	PSQI
Gümüş Şekerci et al. (59)	Turkey	General older adult	60	72.54 ± 8.06	Walking exercise	Non-intervention	40 min/session, 2 session/weeks, 8 weeks	PSQI
Bademli et al. (60)	Turkey	Cognitive impairment	60	71.46 ± 7.75	Walking exercise	Routine activities	80 min/session, 7 session/weeks, 20 weeks	PSQI
Aibar-Almazán et al. (61)	Spain	Postmenopa-usal women	107	68.18 ± 8.35	Pilates	Normal lifestyle	60 min/session, 2 session/weeks, 12 weeks	PSQI
El-Kader and Al-Jiffri (62)	Saudi Arabia	Sedentary older adult	50	65.35 ± 3.98	Aerobic exercise	Normal lifestyle	40 min/session, 3 session/weeks, 6 months	PSG
Pourhabib et al. (63)	Iran	Heart failure	53	67.32 ± 6.61	Walking exercise	Usual care	30 min/session, 3 session/weeks, 12 weeks	PSQI
Curi et al. (64)	Brazil	General older adult	61	64 ± 0.28	Pilates	Monthly meetings	60 min/session, 2 session/weeks, 16 weeks	PSQI
Laredo-Aguilera et al. (66)	Spain	General older adult	38	75.87 ± 5.81	Functional training	Routine activities	60 min/session, 3 session/weeks, 10 weeks	OSQ
Choi and Sohng (65)	Korea	General older adult	63	78.17 ± 5.74	Yoga	Usual care	30–40 min/session, 4 session/weeks, 12 weeks	PSQI
Cramer et al. (67)	Germany	Colorectal cancer	54	68.26 ± 9.69	Yoga	Usual care	90 min/session, 1 session/weeks, 10 weeks	PSQI
Chen et al. (69)	Taiwan	lung cancer	111	63.58 ± 10.65	Walking exercise	Usual care	40 min/session, 3 session/weeks, 12 weeks	PSQI, actigraph
Chen et al. (68)	Taiwan	Mild sleep impairment	63	65.7 ± 0.7	Aquatic exercise	Normal lifestyle	60 min/session, 2 session/weeks, 8 weeks	Actigraph
Sharif et al. (70)	Iran	General older adult	60	64.8 ± 5.2	Aerobic exercise	Routine activities	60 min/session, 3 session/weeks, 12 weeks	PSQI
Taylor-Piliae et al. (71)	America	Stroke survivors	145	69.9 ± 10.0	Tai Chi and strength training	Usual care	60 min/session, 3 session/weeks, 12 weeks	PSQI
Cheung et al. (73)	America	Knee osteoarthritis	36	71.9	Yoga	Normal lifestyle	60 min/session, 1 session/weeks, 8 weeks	PSQI
Irwin et al. (72)	America	Chronic insomnia	73	66.33 ± 7.45	Tai Chi	Healthy education	120 min/session, 1 session/weeks, 4 months	PSG, PSQI, ASI, ESS
Hariprasad et al. (74)	India	Memory complaints	120	75.28 ± 6.89	Yoga	Non-intervention	60 min/day, 6 months	PSQI
Oudegeest-Sander et al. (75)	British	Sedentary older adult	21	69 ± 3	Cycling exercise	Normal lifestyle	45 min/session, 3 session/weeks, 12 months	Actigraph
Nguyen and Kruse (76)	Vietnam	General older adult	96	68.9 ± 5.1	Tai Chi	Routine activities	60 min/session, 2 session/weeks, 6 months	PSQI
Chen et al. (77)	Taiwan	General older adult	55	71.75 ± 8.13	Baduanjin	Non-intervention	30 min/session, 3 session/weeks, 12 weeks	PSQI
Richards et al. (78)	America	General older adult	102	82.03 ± 7.39	Strength training and walking	Usual care	40 min/session, 5 session/weeks, 7 weeks	PSG
McCurry et al. (79)	America	Alzheimer	65	81.69 ± 8.20	Walking exercise	Nondirective dementia care	30 min/session, 4 session/weeks, 2 months	SDI, actigraph
Hosseini et al. (80)	Iran	General older adult	56	69.09 ± 5.37	Tai Chi	Non-intervention	3 session/weeks, 12 weeks	PSQI
Chen et al. (81)	Taiwan	General older adult	128	69.20 ± 6.23	Yoga	Routine activities	70 min/session, 3 session/weeks, 24 weeks	PSQI
Irwin et al. (83)	America	Sleep complaints	112	69.90 ± 6.80	Tai Chi	Health education	40 min/session, 3 session/weeks, 16 weeks	PSQI
King et al. (82)	America	Sleep complaints	66	61.42 ± 6.70	Endurance, strength, and balance exercises	Health education	45 min/session, 5 session/weeks, 12 months	PSG, PSQI, sleep diary
Gary and Lee (84)	America	Heart failure	23	68 ± 12	Walking exercise	Health education	30 min/session, 3 session/weeks, 12 weeks	Sleep diary, actigraph
Frye et al. (85)	America	General older adult	84	69.2 ± 9.26	Tai Chi and low impact exercise	Non-intervention	60 min/session, 3 session/weeks, 12 weeks	PSQI
Singh et al. (86)	America	Depression	28	70.93 ± 1.99	Resistance training	Health education	60 min/session, 3 session/weeks, 10 weeks	PSQI

E, Experimental Group; C, Control Group; PSQI, Pittsburgh Sleep Quality Index; PSG, Polysomnography; ESS, Epworth Sleepiness Scale; ISI, Insomnia Severity Index; OSQ, Oviedo Sleep Questionnaire; ASI, Athens Insomnia Scale; SDI, Sleep Disorders Inventory.

Among the 50 studies included, the exercise interventions varied widely in their approaches, including aerobic exercise, resistance training, interval training, water-based exercises, Tai Chi, Baduanjin, walking, Qigong, yoga, cycling, and Pilates. The duration of the entire intervention period ranged from 4 weeks to 12 months, with a maximum duration of 12 weeks in most studies. The duration of each exercise session also varied, ranging from 30 to 120 min, with the most common duration being 60 min per session. The frequency of intervention per week showed significant differences as well, ranging from once a week to seven times a week, with most interventions occurring three times per week.

In the 39 studies, exercise interventions were compared with no specific treatment, and control conditions included standard care, no intervention, waiting list controls, singing, and prayer. The remaining 11 studies compared exercise interventions with active treatments, such as health education and sleep hygiene education. 34 studies exclusively used subjective measurement scales to assess post-intervention patient-reported sleep outcome. The measurement tools included PSQI, ESS, ISI, AIS, and Oviedo Sleep Questionnaire (OSQ). Five studies solely employed PSG or actigraphy in the measurement of clinician-reported sleep parameters in participants. Additionally, eleven studies combined qualitative and quantitative tools to evaluate participants’ sleep. Overall, the PSQI was the most commonly utilized assessment tool, as it was employed in 41 out of the 50 studies.

### Quality assessment

Table 2 describes the methodological quality assessment of the studies included. Each study met at least four criteria and achieved a moderate quality level or higher. The number of studies with high and moderate quality ratings were 33 and 17, respectively, yielding an average score of 6.06, indicating an overall high level of methodological quality, characterized by transparent participant recruitment criteria, random participant allocation and comprehensive reporting of point measures, variability measures, and between-group statistical results of the key outcomes. Additionally, the groups were comparable at baseline for the most important prognostic indicators. More than two-thirds of the studies included maintained a high retention rate during the intervention period. However, due to the limitations of exercise interventions, blinding of participants and therapists was not implemented in most studies.

**Table 2:** Quality assessment of included studies.

Study (year)	EC	RA	CA	SAB	SB	TB	AB	DR	ITA	BC	PM	TS	OSQ
Wang et al. (39)	1	1	1	1	0	0	0	1	0	1	1	6	High
Song et al. (40)	1	1	1	1	1	0	0	0	1	1	1	7	High
Sánchez-Alcalá et al. (41)	1	1	1	1	0	0	0	1	0	1	1	6	High
Li et al. (42)	1	1	1	1	0	0	1	1	0	1	1	7	High
He et al. (43)	1	1	0	1	0	0	1	1	1	1	1	7	High
Teruel-Hernández et al. (44)	1	1	0	1	1	0	0	1	1	1	1	7	High
Tung et al. (45)	1	1	0	1	0	0	0	1	0	1	1	5	Moderate
Baklouti et al. (46)	1	1	0	1	0	0	0	0	0	1	1	4	Moderate
Zhou et al. (47)	1	1	1	1	1	0	0	1	0	1	1	7	High
de Sá Souza et al. (27)	1	1	0	1	0	0	1	1	1	1	1	7	High
Song et al. (48)	1	1	1	1	0	0	1	1	1	1	1	8	High
Marupuru et al. (28)	1	1	1	1	0	0	1	1	1	1	1	8	High
Siu et al. (49)	1	1	1	1	0	0	1	1	1	1	1	8	High
Li et al. (50)	1	1	0	1	0	0	1	1	0	1	1	6	High
Shree Ganesh et al. (51)	1	1	1	1	0	0	0	0	1	1	1	6	High
de Lima et al. (52)	1	1	1	1	0	0	0	0	0	1	1	5	Moderate
Jiménez-García et al. (53)	1	1	0	1	1	1	1	1	0	1	1	8	High
Wang et al. (54)	1	1	1	1	0	0	1	1	0	1	1	7	High
Tseng et al. (55)	1	1	1	1	0	0	1	0	0	1	1	6	High
Phansuea et al. (56)	1	1	0	1	0	0	0	1	1	1	1	6	High
Fan et al. (57)	1	1	0	1	0	0	0	0	1	1	1	5	Moderate
Song et al. (58)	1	1	1	1	0	0	1	1	1	1	1	8	High
Gümüş Şekerci et al. (59)	1	1	0	1	0	0	0	1	0	1	1	5	Moderate
Bademli et al. (60)	1	1	1	1	0	0	0	1	1	1	1	7	High
Aibar-Almazán et al. (61)	1	1	1	1	0	0	1	1	0	1	1	7	High
El-Kader and Al-Jiffri (62)	1	1	0	1	0	0	0	1	1	1	1	6	High
Pourhabib et al. (63)	1	1	0	1	0	0	0	0	0	1	1	4	Moderate
Curi et al. (64)	1	1	0	1	0	0	0	1	0	1	1	5	Moderate
Laredo-Aguilera et al. (66)	1	1	0	1	0	0	0	1	0	1	1	5	Moderate
Choi and Sohng (65)	1	1	0	1	0	0	1	0	0	1	1	5	Moderate
Cramer et al. (67)	1	1	1	1	0	0	0	0	1	1	1	6	High
Chen et al. (69)	1	1	0	1	0	0	0	1	0	1	1	5	Moderate
Chen et al. (68)	1	1	0	1	0	0	0	0	0	1	1	4	Moderate
Sharif et al. (70)	1	1	0	1	0	0	0	1	1	1	1	6	High
Taylor-Piliae et al. (71)	1	1	0	1	0	0	1	1	1	1	1	7	High
Irwin et al. (72)	1	1	0	1	0	0	1	0	1	1	1	6	High
Cheung et al. (73)	1	1	0	1	0	0	1	1	1	1	1	7	High
Hariprasad et al. (74)	1	1	0	1	0	0	1	0	1	1	1	6	High
Oudegeest-Sander et al. (75)	1	1	0	1	0	0	0	1	0	1	1	5	Moderate
Nguyen and Kruse (76)	1	1	0	1	0	0	0	1	1	1	1	6	High
Chen et al. (77)	1	1	0	1	0	0	0	1	0	1	1	5	Moderate
Richards et al. (78)	1	1	1	1	0	0	0	1	1	1	1	7	High
McCurry et al. (79)	1	1	1	1	0	0	1	0	0	1	1	6	High
Hosseini et al. (80)	1	1	0	1	0	0	0	1	0	1	1	5	Moderate
Chen et al. (81)	1	1	0	1	0	0	0	1	0	1	1	5	Moderate
King et al. (82)	1	1	0	1	0	0	1	1	1	1	1	7	High
Irwin et al. (83)	1	1	1	1	1	0	0	1	1	1	1	8	High
Gary et al. (84)	1	1	0	1	0	0	0	0	0	1	1	4	Moderate
Frye et al. (85)	1	1	0	1	0	0	0	0	0	1	1	4	Moderate
Singh et al. (86)	1	1	0	1	0	0	1	1	0	1	1	6	High
Mean score												6.06	

Yes = 1; No = 0. EC, eligibility criteria; RA, random allocation; CA, concealed allocation; SAB, similar at baseline; SB, subject blinded; TB, therapist blinded; AB, assessor blinded; DR, dropout rate (< 15%); ITA, intention-to-treat analysis; BC, between-group comparison; PM, points measures; TS, total score; OSQ, overall study quality.

### Patient-reported sleep parameters

**Figure 2:**
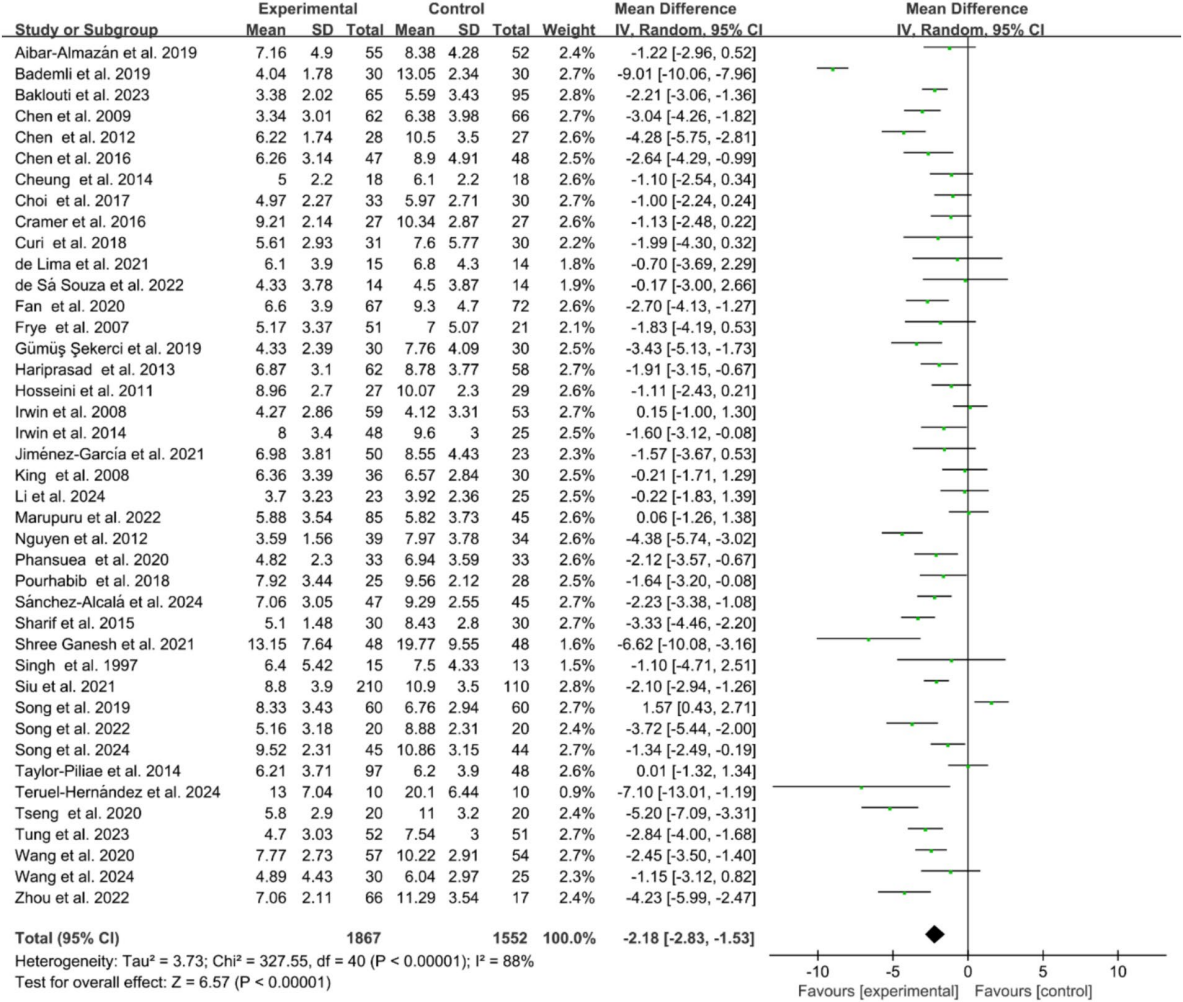
Forest plot of the effects of exercises on PSQI total score.

**Figure 3:**
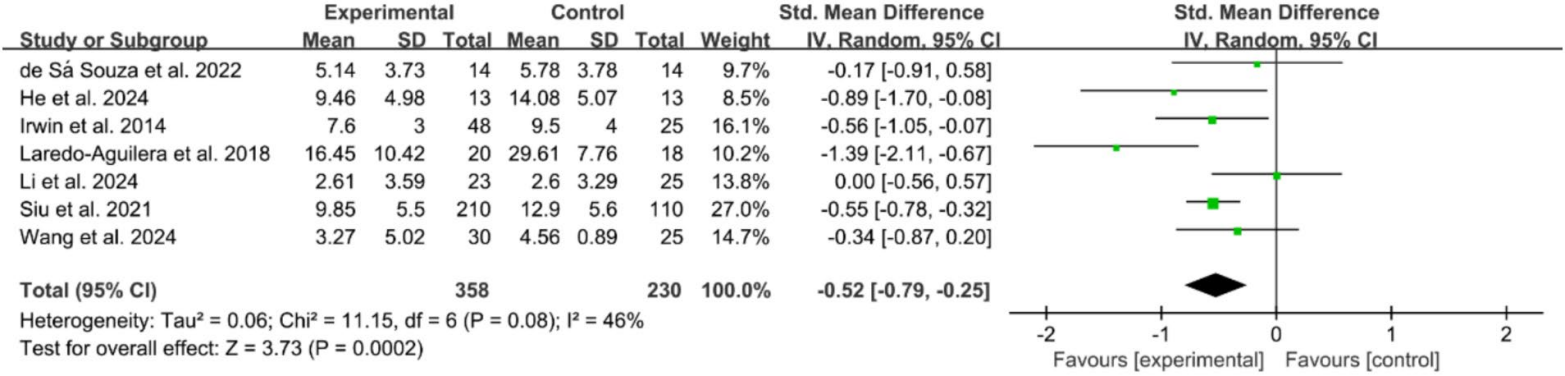
Forest plot of the effects of exercises on insomnia.

**Figure 4:**
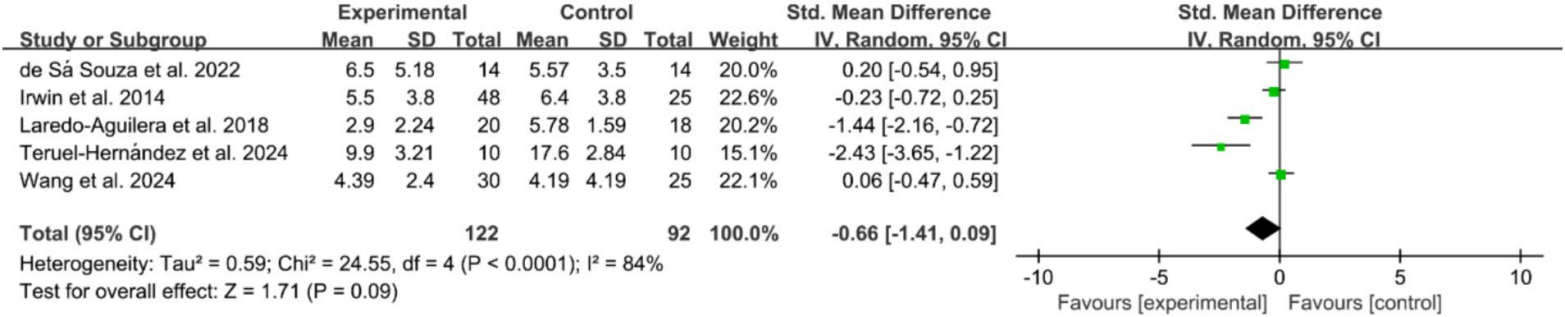
Forest plot of the effects of exercises on daytime sleepiness.

Of the included studies, 41 reported the post-intervention PSQI total score of the participants. The PSQI is primarily used in clinical and basic research to assess subjective sleep quality, with a total score ranging from 0 to 21, where higher scores indicate poorer sleep quality. Figure 2 presents a forest plot of the difference in PSQI total scores between the experimental and control groups. Due to an *I^2^* = 88%, a random-effects model was used for calculation, indicating a significantly beneficial effect of exercises on sleep quality as reflected by the PSQI total score (WMD = −2.18, 95%CI: −2.83 to −1.53, *p* < 0.01). Additionally, seven studies reported the post-intervention severity of insomnia among participants, using assessment tools such as ISI, AIS, and OSQ, which are tools utilized for self-reported insomnia severity measurement, where higher scores indicate more severe insomnia. Figure 3 shows a forest plot of the difference in insomnia severity between the experimental and control groups. Using a random-effects model, the results indicated a notable reduction in insomnia severity in the experimental group compared to the control group (SMD = −0.52, 95%CI: −0.79 to −0.25, *p* < 0.01). Besides, five studies reported the daytime sleepiness of participants post-intervention. Figure 4 presents a forest plot of the difference in daytime sleepiness, showing no significant difference between the experimental and control groups (SMD = −0.66, 95%CI: −1.41 to 0.09, *p* = 0.09).

### Clinician-reported sleep parameters

**Figure 5:**
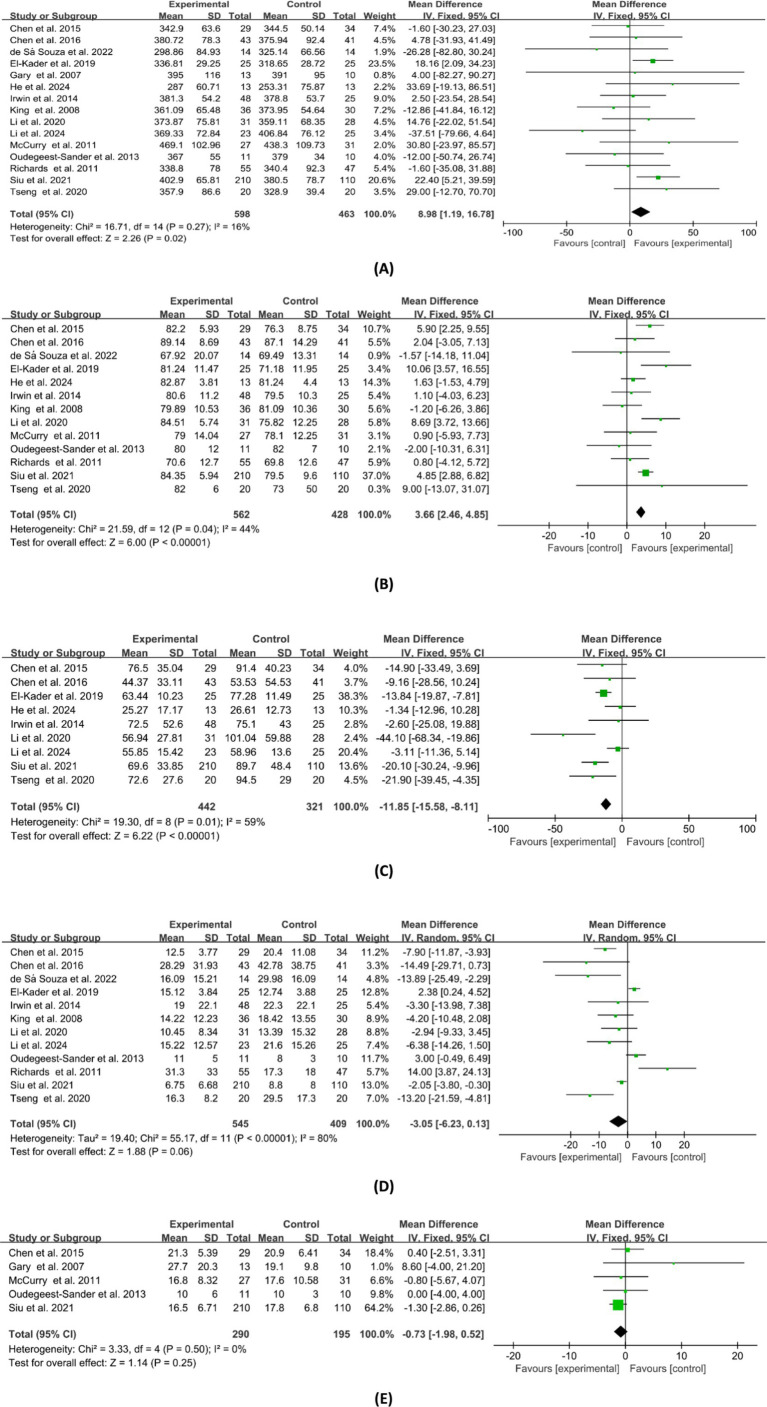
Forest plot of the effects of exercises on clinician-reported sleep parameters. **(A)** TST; **(B)** SE; **(C)** WASO; **(D)** SL; **(E)** no. of awakenings.

Regarding clinician-reported sleep parameters, participants’ total sleep time (TST), sleep latency (SL), sleep efficiency (SE), wake after sleep onset (WASO), and the number of awakenings before and after the intervention were measured in the studies utilizing PSG or actigraphy. Figure 5 depicts the combined effect sizes of these clinician-reported sleep parameters. Fifteen studies reported TST of pre-intervention and post-intervention, and the combined results showed a significant difference between the experimental and control groups (WMD = 8.98, 95%CI: 1.19 to 16.78, *p* < 0.05). Thirteen studies compared changes in pre-intervention and post-intervention SE between the experimental and control groups, with combined results indicating a positive effect of exercise interventions on SE (WMD = 3.66, 95%CI: 2.46 to 4.85, *p* < 0.01). Combining results from nine studies reporting WASO outcomes revealed that the experimental group had significantly less WASO compared to the control group (WMD = −11.85, 95%CI: −15.58 to −8.11, *p* < 0.01). However, no significant differences were found between the experimental and control groups in terms of SL (WMD = −3.05, 95%CI: −6.23 to 0.13, *p* = 0.06) and the number of awakenings (WMD = −0.73, 95%CI: −1.98 to 0.52, *p* = 0.25).

### Sensitivity analysis

The sensitivity analysis conducted on the combined results of patient-reported sleep parameters demonstrated that the exclusion of any single study did not lead to significant changes in the combined effect size and 95% confidence intervals (CIs). This indicates a high level of stability in the analysis results for patient-reported sleep parameters. Regarding the sensitivity analysis of combined results for clinician-reported sleep parameters, the analyses for SE, WASO, and the number of awakenings were found to be stable. However, variability was noted in TST and SL. Specifically, after the exclusion of the study by Siu et al., the combined result for TST changed substantially from (WMD = 8.98, 95%CI: 1.19 to 16.78, *p* < 0.05) to (WMD = 5.51, 95%CI: −3.24 to 14.25, *p* = 0.22). Similarly, after excluding the study by Richards et al., the combined result for SL shifted from (WMD = −3.05, 95%CI: −6.23 to 0.13, *p* = 0.06) to (WMD = −3.94, 95%CI: −7.02 to −0.86, *p* < 0.05). Therefore, caution should be exercised in the interpretation of the TST and SL results.

### Subgroup analysis

This study conducted subgroup analysis on the meta-analysis results for PSQI total score, TST, SE, and WASO. Table 3 depicts the subgroup meta-analysis results based on participant source, control type, and study quality. No significant differences were observed among the indicators based on participant source and study quality. As for control type, significant intergroup differences were found in SE (*p* < 0.05). Besides, only when the controlled conditions were non-active treatment, the combined results of TST and SE were of statistical significance.

**Table 3:** Effects of exercise intervention on sleep with different moderators.

Outcome	Category of variables	Studies (*n*)	Participants (*n*)	WMD (95% CIs)	*p*-value (overall effect)	*I^2^* value (%)	*p-*value (heterogeneity)	*p*-value (group differences)
PSQI	Source of participants							
	General population	17	1,236	−2.49 (−3.21 to −1.77)	<0.01	71	<0.01	0.39
	Patients	24	2,183	−1.96 (−2.93 to −0.99)	<0.01	91	<0.01	
	Type of control							
	Active treatment	10	699	−1.69 (−3.01 to −0.38)	0.01	86	<0.01	0.42
	Usual care or placebo	31	2,720	−2.32 (−3.05 to −1.59)	<0.01	87	<0.01	
	Quality of the study							
	Moderate quality	13	1,074	−2.35 (−2.89 to −1.81)	<0.01	42	0.05	0.70
	High quality	28	2,345	−2.14 (−3.06 to −1.21)	<0.01	91	<0.01	
TST	Source of participants							
	General population	5	261	8.60 (−3.74 to 20.93)	0.17	52	0.08	0.94
	Patients	10	800	9.24 (−0.82 to 19.29)	0.07	0	0.50	
	Type of control							
	Active treatment	5	260	4.28 (−12.15 to 20.70)	0.61	0	0.46	0.52
	Usual care or placebo	10	801	10.35 (1.49 to 19.20)	<0.05	29	0.18	
	Quality of the study							
	Moderate quality	4	191	−2.12 (−21.15 to 16.90)	0.83	0	0.94	0.21
	High quality	11	870	11.22 (2.67 to 19.76)	<0.05	32	0.14	
SE	Source of participants							
	General population	4	213	3.20 (−0.30 to 6.71)	0.07	57	0.07	0.79
	Patients	9	777	3.72 (2.45 to 4.99)	<0.01	45	0.07	
	Type of control							
	Active treatment	4	237	0.32 (−2.83 to 3.48)	0.84	0	0.79	<0.05
	Usual care or placebo	9	753	4.22 (2.93 to 5.51)	<0.01	48	0.05	
	Quality of the study							
	Moderate quality	3	168	3.85 (1.05 to 6.64)	<0.01	44	0.17	0.88
	High quality	10	822	3.62 (2.29 to 4.94)	<0.01	50	<0.05	
WASO	Source of participants							
	General population	3	138	−10.95 (−15.64 to −6.26)	<0.01	66	0.05	0.54
	Patients	6	625	−13.39 (−19.55 to −7.23)	<0.01	62	<0.05	
	Type of control							
	Active treatment	2	113	−14.60 (−28.43 to −0.76)	<0.05	43	0.18	0.69
	Usual care or placebo	7	650	−11.63 (−15.51 to −7.76)	<0.01	65	<0.01	
	Quality of the study							
	Moderate quality	2	147	−12.15 (−25.58 to 1.27)	0.08	0	0.68	0.96
	High quality	7	616	−11.82 (−15.71 to −7.94)	<0.01	69	<0.01	

PSQI, Pittsburgh Sleep Quality Index; TST, Total sleep time; SE, Sleep efficiency; WASO, Wake after sleep onset.

### Publication bias

**Figure 6:**
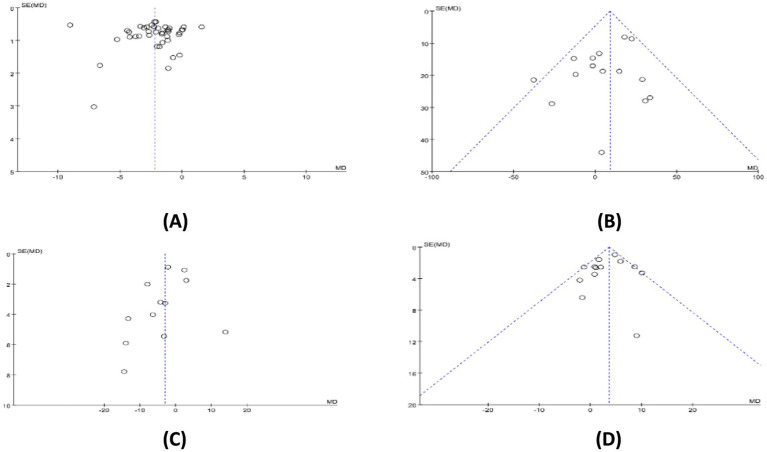
The funnel plot for publication bias. **(A)** PSQI total score; **(B)** TST; **(C)** SL; **(D)** SE.

Since the meta-analyses for PSQI total score, TST, SL, and SE included more than 10 studies each, we investigated the publication bias in them. Figure 6 shows that most studies are positioned above the center line, and the distribution is roughly symmetrical on both sides. Additionally, Egger’s test *p*-values for these analyses were 0.978, 0.160, 0.241, and 0.410, respectively, all of which are greater than 0.05. In summary, these results suggest no notable publication bias in the studies included in each of these meta-analyses.

## Discussion

Sleep is a complex physiological phenomenon, whose quality should be assessed by integrating subjective sleep perceptions and clinician-reported sleep parameters (87). This study conducted a systematic review and meta-analysis of RCTs with the highest-level evidence to summarize the effectiveness of exercise interventions in the improvement of sleep quality among older adult population. The results of the study indicate a positive impact of exercise interventions on sleep among older adult people. This positive impact is specifically manifested in three aspects: First, a significant decrease in overall PSQI scores reflects improved subjective sleep quality. Second, after exercise interventions, self-reported severity of insomnia is effectively alleviated. Finally, in terms of clinician-reported sleep outcomes, improvements in TST, SE, and WASO with exercise interventions show clinical relevance compared to active treatments, routine care, or waitlist controls. To our knowledge, there is currently no meta-analysis comprehensively assessing the impact of physical exercise on clinician-reported sleep outcomes in older adult population. Therefore, our study has provided more comprehensive and robust evidence in the summary and confirmation of the true significance of exercise on sleep among elder individuals.

The PSQI is a classic subjective sleep quality assessment tool widely used in epidemiological studies of sleep disorders. In our Meta-analysis, the PSQI total score showed a change of −2.18 points, which was of statistical significance (88). Regarding the minimal clinically important difference (MCID) for the PSQI, studies suggest a range between 1.14 to 1.80. The score change we observed aligns closely with the two recent meta-analyses. In the study by Solis-Navarro et al., older adult people in the exercise intervention group showed a decrease of 2.49 in PSQI scores compared to the control group, with merely 8 studies included in their analysis (89). Wu et al.’s meta-analysis demonstrated a significant decrease of 2.34 in PSQI scores among older adult people participating in traditional Chinese exercises or general aerobic exercises compared to control conditions (90). Compared to these studies, our research included more high-quality RCTs and encompassed a broader range of intervention measures. Our study, therefore, further confirms that exercise interventions obtain clinically meaningful benefits for subjective sleep quality in older adult people. Currently, there are several explanations for the mechanisms underlying the relationship between exercise and sleep quality. One viewpoint suggests that elevated levels of pro-inflammatory cytokines can worsen insomnia, while exercise can help restore a stable sleep–wake cycle by improving TNF-*α*, IL-1β, IL-6, and other pro-inflammatory cytokines (91, 92). Other proposed mechanisms include increasing exposure to sunlight, enhancing metabolic capacity, alleviating stress and anxiety, and regulating temperature changes (75, 93–95). It’s important to note that our analysis revealed a high degree of heterogeneity in PSQI scores among the studies (*I^2^* = 88%). However, subgroup meta-analysis based on participant source, control type, and methodological quality showed no significant inter-group differences. Given the varied nature of exercise interventions included in our study, we speculate that this may be an important factor contributing to the observed heterogeneity.

Moreover, this study delved into the impact of exercise intervention on the severity of insomnia and daytime sleepiness. According to Cohen’s recommended effect size criteria (96), exercise intervention demonstrated a moderate effect size (*d* = −0.52) on reducing insomnia severity in the older adult, consistent with previous research focusing on adults clinically diagnosed with insomnia (97). Therefore, it appears that the influence of exercise on insomnia severity is not affected by age. Our study results suggest that exercise does not have a significant positive impact on daytime sleepiness in older adult people. Some studies suggest that the effect of exercise on daytime sleepiness depends on the timing of exercise, with benefits observed when exercise is performed in the morning (98). After waking up in the morning, the cerebral cortex is in an inhibited state, and moderate exercise can increase cortical excitability, thereby reducing daytime sleepiness (99). However, our meta-analysis on daytime sleepiness was based on merely five studies, none of which reported the timing of exercise. Therefore, it is challenging to determine whether our results are related to the timing of interventions included in the studies. Future research should include more high-quality trials to confirm the impact of exercise on daytime sleepiness in older adult people and investigate on whether the timing of exercise plays a regulatory role.

Older adult individuals may exhibit biases in self-assessment of their sleep conditions, emphasizing the need for objective measurements to validate these assessments (87). To comprehensively evaluate the effectiveness of exercise on sleep in older adult people, we analyzed clinician-reported parameters measured via PSG or actigraphy, including TST, SE, SL, WASO, and the frequency of awakenings. The results demonstrate that exercise has beneficial effects on many clinician-reported sleep indicators in older adult people. Consistent with previous research (89), we found that exercise positively impacts SE among this demographic cohort. However, there is rather limited understanding of exercise’s effect on SE. A recent survey revealed that pain, nocturia, and sleep medication use are closely associated with SE in older adult people (100), with those using sleep medications more likely to have lower SE (13). Previous evidence suggests that regular physical exercises can alleviate chronic pain in older adult population, as well as reduce nocturia, and decrease sleep medication use (101–103). The beneficial effects of exercise on these influencing factors may represent potential mechanisms explaining the improvement in SE in older adult people. Additionally, it is found that exercise positively influences TST and WASO in older adult people, which has not been reported previously. This finding is of great significance in that these factors are key diagnostic indicators of sleep disorders and are related to increased mortality in older adult people (104). In this study, we observed that exercise did not reduce SL or the number of awakenings. Frequency of awakenings refer to the number of awakenings lasting 1 min or above during the night of sleep, excluding the last awakening before waking up (105). Some studies suggest that baseline physical activity levels are an important moderating variable affecting the relationship between exercise and the number of awakenings in older adult people, with greater improvements observed in older sedentary individuals (106). However, most of the 5 studies included in our analysis did not report participants’ baseline physical activity levels, making it difficult to determine whether our results are related to this variable. It is important to note that sensitivity analysis revealed unstable results for TST and SL, indicating significant potential bias factors related to exercise intervention efficacy that require further investigation for validation.

Furthermore, in our subgroup analysis based on control types, insightful revelations have been discovered. The combined effect sizes of TST and SE proved statistically significant in the non-active treatment subgroup, but not in the active treatment subgroup. The active treatment subgroup included control conditions such as sleep hygiene education or health promotion courses, indicating they may have some positive impact on sleep in older adult people. Limited evidence suggests that sleep hygiene education interventions lead to significant improvements in sleep complaints or insomnia in patients, achieving small to moderate effect sizes (107). Future research could explore the impact of combined exercise interventions with sleep hygiene education on sleep among the older adult.

This review is beset by several limitations. Firstly, some studies exhibited high heterogeneity among them, therefore our results should be interpreted with caution. Subgroup analyses based on participant sources, control types, and methodological quality inadequately explicate the origins of this heterogeneity. Future investigations need additional moderating variables for grouping and analysis, such as gender, physical activity levels, types of exercise, exercise intensity, and exercise duration. Secondly, most studies did not implement blinding of participants and therapists, which could potentially exaggerate the current findings. Lastly, we solely included studies published in English in peer-reviewed journals. Future endeavors could incorporate broader literature searches and the inclusion of relevant studies from a more extensive range of sources.

## Conclusion

Our research findings indicate that exercise has a beneficial effect on enhancing sleep in older adult people, offering a safe and effective approach. Following exercise intervention, older adult individuals reported significant improvements in subjective sleep quality and reductions in insomnia severity, along with positive impacts on objective measures such as TST, SE, and WASO. It not only provides evidence-based support for the formulation of exercise prescriptions and health management policies, but also offers references for other researchers engaged in this field.

## Data Availability

The original contributions presented in the study are included in the article/Supplementary material, further inquiries can be directed to the corresponding author.

## Footnotes

^1^ https://www.crd.york.ac.uk/prospero/
